# The Feasibility of Telemedicine in the Implementation and Management of Therapeutic Hypothermia for Infants with Neonatal Hypoxic-Ischemic Encephalopathy in a Resource-Limited Country

**DOI:** 10.1055/s-0042-1760434

**Published:** 2023-02-23

**Authors:** Adnan Hadid, Taher S. AL-Shantout, Rayan S. Terkawi, Baraa M. Aldbes, Manal M. Zahran, Fadia A. Alsatouf, Hani Najjar, MHD Hassan Mughrabieh, Nour A. Alhadid, Khalid Altirkawi

**Affiliations:** 1 Department of Pediatric, College of Medicine, King Saud University, Riyadh, Saudi Arabia; 2 Neonatal Intensive Care Unit, King Saud University Medical City, Riyadh, Saudi Arabia; 3 Department of Pediatric, Ohud Hospital, MOH, Madinah, Saudi Arabia; 4 Neonatal Intensive Care Unit, Ohud Hospital, MOH, Madinah, Saudi Arabia; 5 Department of Pediatrics, Hamad General Hospital, Doha, Qatar; 6 Department of Pediatrics, Sidra Medicine, Doha, Qatar; 7 Department of Pediatrics, Maternity and Children Hospital, Najran, Saudi Arabia; 8 Department of Pediatrics, EL-Ekhaa Hospital, Syrian Expatriate Medical Association, Idlib, Syria; 9 Department of Pediatrics, Aladan Hospital, Hadiya, Kuwait; 10 Syrian Expatriate Medical Association (SEMA), Gaziantep, Turkey; 11 College of medicine, Alfaisal University, Riyadh, Saudi Arabia

**Keywords:** hypoxic-ischemic encephalopathy, therapeutic hypothermia, cooling therapy, NICU, telemedicine

## Abstract

**Background** Telemedicine is widely used in neonatal services in developed countries, though its outcomes in low- and middle-income countries are controversial. Lack of expertise and/or facilities, however, has limited its use in developing countries and around areas of military conflicts. We aim to study the implementation and management of therapeutic hypothermia (TH) in infants with hypoxic-ischemic encephalopathy (HIE) with the help of telemedicine in a resource-limited country.

**Methodology** This is a retrospective study, evaluating patients who received TH, guided by telemedicine, through a mobile app (Telegram), an application that allows sharing and archiving of information with other beneficial features. We assessed the feasibility of utilizing telemedicine in guiding the application of TH to infants affected with HIE in the North-West of Syria between July 2020 and July 2021. Feasibility was measured by parameters related to the time gaps between initiation of consultation and treatment and clinical short-term outcomes.

**Results** Out of 5,545 newborn infants delivered during the study period, 22 patients were eligible for TH guided by telemedicine. Patients were referred for consultation at a median (interquartile range [IQR]) of 137 (35–165) minutes of life. A median (IQR) of 12 (3–18) minutes elapsed between the call for a consultation and the consultant response and a median (IQR) of 30 (0–42) minutes elapsed between seeking the consultation and the initiation of cooling therapy. Eighteen patients completed cooling for 72 hours. The patients' temperatures were within the target range (33–34°C) most of the time (84.1%).

**Conclusion** Telemedicine is a feasible method to guide the implementation TH for HIE in resource-limited areas. The short-term success rate is relatively high; however, further studies with a larger population are needed to confirm these findings.

## Introduction

Hypoxic-ischemic encephalopathy (HIE) is a leading cause of morbidity and mortality worldwide. In 2010, HIE incidence was estimated to be 8.5 cases per 1,000 live births; the majority of cases (96%) were born in low- and middle-income countries (LMICs). 1 If survived the neonatal period, affected infants are likely to experience an increased burden of neurodevelopmental impairments. Therefore, HIE is not just a disease of the neonatal period but far beyond. 2

Obstacles to the implementation of therapeutic hypothermia (TH) in low-income countries are not limited to the lack of equipment; neonatal intensive care units (NICU) may lack necessary expertise too. Direct threats to medical care professionals and the killing of many of them led to the displacement of others. These shortages in providers added to the destruction of many facilities and lack of essential equipment, which left the whole healthcare system with major deficiencies, 3 4 especially in terms of delivering tertiary care for the most vulnerable populations, such as infants with HIE. 5 Providing quality medical education to the providers who are able to stay and handle the burden was a challenge that was difficult, to say the least.

To fill some of the critical gaps, telemedicine was utilized; using continuous conference video calls was described in level II NICU in underserved areas in the United States, which showed its effectiveness and safety. 6 Telemedicine in HIE management was reported by Craig et al. 7 They used Cisco conferencing video calling software to connect neonatologists with pediatric neurologists. The technologies mentioned, their cost, and the need for infrastructures to operate them, such as high-speed Internet, make them inapplicable in certain circumstances, such as conflict areas in northeast Syria, where more creative methods of telemedicine are needed.

The primary outcome is to emphasize the feasibility of implementing TH through telemedicine, measured by parameters related to telemedicine success in implementing TH in a timely and efficient manner.

## Methods and Settings

### Study Design, Participants, and Settings

This is a retrospective, single-center cohort study conducted in El-Ekhaa specialized hospital, a maternity and children hospital in an area ravaged by military conflict in the North-West of Syria, between July 2020 and July 2021. The hospital is located in the middle of dozens of refugee camps, which accommodate approximately 600,000 refugees. It offers both inpatient and outpatient pediatric and gynecological services. In addition to antenatal care clinics and performing normal deliveries, the hospital services include instrumental and cesarian deliveries and both major and minor gynecological surgeries. The pediatric services include a 25-bed general inpatient service, a three-bed pediatric intensive care unit, in addition to the NICU equipped with 10 incubators and conventional and noninvasive mechanical ventilation devices. The NICU offers a wide range of neonatal services, such as phototherapy, exchange transfusion, and surfactant replacement therapy. The hospital also has an emergency department that serves women's and children's emergencies around the clock. It is an academic and research center in the region that previously published on children's and women's health. 8 9 The hospital has no computerized tomography (CT) or magnetic resonance imaging (MRI) machines. Therefore, patients who had those imaging studies had them after discharge. An ultrasound machine was added to the hospital later on, and operated by NICU medical personnel after receiving certified training. Advanced cooling devices were not available. An electroencephalography machine was not available as well.

Calgary University's HIE calculator was used to facilitate treatment decisions. This tool has been used by the primary medical provider at the bedside and its accurate application was confirmed by the neonatologist on-call via Telemedicine. ( https://play.google.com/store/apps/details?id=com.radsun.hiecalculator&hl=en_CA&gl=US )

Patients were cooled if they were more than or equal to 35 weeks and a birth weight more than or equal to 1800 g with evidence of perinatal depression as indicated by the presence of either one of the following (criteria A):

(i) pH less than or equal to 7 or base excess less than or equal to -16 on cord blood gas analysis or one performed within 1 hour after delivery; or
(ii) The need for positive pressure ventilation for more than or equal to 10 minutes, or Apgar score less than or equal to 5 at 10 minutes.

With the presence of (criteria B): HIE stage II or III according to modified Sarnat classification, 10 or convulsion.

Patients were excluded if they had severe congenital malformations, high oxygen requirement (fraction of inspired oxygen ≥ 90%), or severe coagulopathy with uncontrolled bleeding.

The treating team consisted of in-hospital pediatricians with an online consulting team that includes three neonatologists providing around-the-clock coverage, a radiologist, and other pediatric subspecialists, including a pediatric neurologist, nephrologist, and hematologist.

Based on Calgary tool criteria, if a patient is deemed eligible, the case was discussed with the online on-call neonatologist. Detailed maternal and perinatal histories with all available investigations, including the radiographs, were forwarded through a mobile app (Telegram). Telegram has the ability to keep all records with no limitations. New members of the group will be able to reach old records. Uploading documents in big sizes is possible with Telegram for up to 2 TB, which is essential when long videos are shared.

When the decision of cooling was made (by the on-call neonatologist together with the primary pediatrician), the passive cooling process was initiated; the radiant warmer was switched off, followed by transferring the patient to an incubator. The neonatal care unit room temperature was adjusted using air conditioners, but a room temperature monitor was not available at that time. During the winter season, setting the temperature of the incubator at 30°C seemed a good starting point in our practice. On the other hand, incubators were set off during the summer season. This approach was modified, based on the discretion of the treating physician and on case-to-case bases. Temperatures were readjusted frequently during the initiation phase based on skin and core temperatures. However, all recorded temperatures reported in this study were the rectal temperatures, whether using a continuous monitor or an hourly inserted rectal thermometer when the continuous monitor was not yet available. Bedside nurses were charting temperatures on hourly bases. Later, the temperature was adjusted as needed to keep the infant's core temperature within the target range (33–34°C). Morphine was administered as an adjunct therapy to decrease pain and achieve better control of the temperature, using a continuous infusion pump, at a rate of 20 µg/kg/hour. When available, a rectal probe was used for continuous monitoring of core temperature; otherwise, a skin probe was used for this purpose, with hourly determinations of core (rectal) temperature using a mercury thermometer. In some cases, if the temperature remained out of range, further measures were taken, including ice bags and adjusting room temperature.

Laboratory investigations performed on all infants included complete blood count), hepatic and renal function tests, prothrombin time), activated partial thromboplastin time, and C-reactive protein. Due to the lack of microbiology laboratory facility, blood cultures were not performed in any of these infants. However, empiric antibiotics (ampicillin and cefotaxime) were used in all of them due to the tentative diagnosis of “clinical sepsis” and were broadened in case of clinical deterioration.

Videos of the baby upon admission and later, if she/he developed convulsion or any abnormal movement, were recorded and forwarded to the neonatologist and pediatric neurologist through the mobile application (Telegram). Treating the convulsions and the duration of therapy were based on pediatric neurologist's recommendations. Brain CT or MRI scans were also sent to the radiologist for reading through telemedicine.

### Outcome Measures

Primary outcomes included the age at the consultation requested, the time for the neonatologist's response, and the time lapse between the referral and the point of cooling initiation were recorded. Telegram history was utilized to extracted these data.

Secondary outcomes included short-term clinical outcomes—duration of respiratory support, time to reach full feeding, and survival to discharge.

As developmental pediatrics specialty was not available, all cases were given an appointment for follow-up in the general pediatric clinic to assess development.

### Statistical Analysis

We performed descriptive statistics on the total cohort of patients without stratification. Analysis was performed using Excel software (Microsoft Company, Seattle, Washington, United States). As the data were non-normally distributed, we reported numerical variables as median and interquartile ranges (IQRs). Categorical variables were reported as percentages. No meaningful comparisons within the cohort were possible due to the small sample size.

## Results

During the study period, from July 2020 to July 2021, 5,545 pregnant women were delivered in El-Ekhaa Hospital; of them, 1290 (23.3%) were cesarian section deliveries.

Table 1 presents the patients' characteristics, perinatal management, and cooling process variables. Twenty-two babies were eligible for cooling, 18 (82%) of them had completed cooling for 72 hours, and 4 (18%) babies were excluded. Four patients were excluded from analysis because the cooling protocol was not fully implemented; one patient developed pulmonary hypertension and severe respiratory distress syndrome that required inhaled nitric oxide and more advanced care, thus he was transferred abroad to Turkey. The second patient failed to achieve the cooling target despite using the aforementioned methods. Two patients were excluded because cooling was initiated at the age of 8 hours in one of them, and the other was rewarmed at the age of 2 days. As this study is retrospective, the clinical decision led to those actions that were acknowledged and the patients were excluded from the analysis.

**Table 1:** Patients' characteristics, perinatal management, and cooling process

*n*	18
Gestational age, weeks; median (IQR)	38 (38–40)
Birthweight, grams; median (IQR)	3,400 (3,200–3,500)
Male, *n* (%)	13 (72)
Primigravida; *n* (%)	8 (44)
Vaginal delivery; *n* (%)	17 (94)
Meconium-stained amniotic fluid; *n* (%)	7 (39)
Resuscitation initiated by a doctor; *n* (%)	3 (17)
Apgar's score at 1; median (IQR)	3 (3–3.5)
Apgar's score at 5; median (IQR)	5 (4–5.25)
Apgar's score at 10; median (IQR)	6 (5–6)
PPV > 10 minute; *n* (%)	15 (83)
Chest compressions; *n* (%)	1 (6)
Cord blood gases; *n* (%)	5 (28)
Arterial blood gases in 1h; *n* (%)	11 (61)
pH; median (IQR)	7.09 (7.07–7.18)
Base deficit; median (IQR)	21 (17 -25)
Serum HCO _3_ ; median (IQR)	8.1 (5.7–9.6)
Age when TH was initiated, hours; of life; median (IQR)	1 (1–2)
Time to achieve target temperature, hours; median (IQR)	1 (1–3)
Continuous rectal sensor used (to regulate temperature); *n* (%)	10 (56)
Stage 2 of encephalopathy; *n* (%)	17 (94)
Progressed to stage 3 of encephalopathy; *n* (%)	1 (6)
Had clinical convulsions, *n* (%)	15 (83)
Had clinical convulsions before 6h of age; *n* (%)	3 (17)
Anti-seizure drugs used, %	14 (78)

Abbreviations: HCO3, Bicarbonate; IQR, interquartile range; N, number; PPV, positive pressure ventilation; TH, therapeutic hypothermia.

**Fig. 1:**
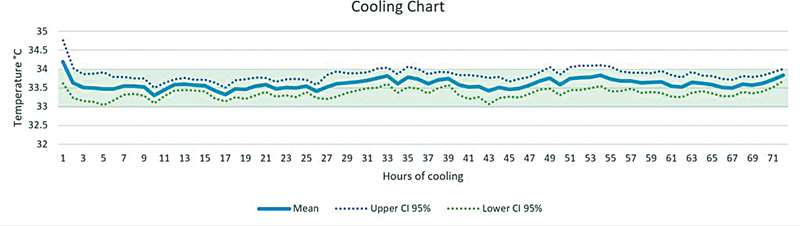
The mean and 95% CI (confidence interval) of temperature readings during therapeutic hypothermia for the total cohort of patients.

Notably, patients' temperatures remained within the target range (33–34°C) most of the time (84.1% of the temperature readings), 5% of these readings were less than 32°C, and 11% of them were above 34°C ( Fig. 1 ). Of 18 patients, 12 patients (66.7%) achieved target range in the first hour of therapy, four in the second hour, one in the fourth hour, and one in the fifth hour.

Twenty-two patients were referred through telemedicine for TH eligibility. Records of these patients were analyzed. Patients were referred for consultation at a median (IQR) of 137 (35–165) minutes of life. A median (IQR) of 12 (3–18) minutes elapsed between the call for a consultation and the consultant's response, and a median (IQR) of 30 (0–42) minutes elapsed between seeking the consultation and the initiation of cooling therapy. All the patients who completed TH were followed daily through telemedicine till discharge.

Most of the deliveries were vaginal; only one patient (6%) was delivered by C-section. Fifteen out of the 18 (83%) patients required positive pressure ventilation for more than 10 minutes, and one patient (6%) required chest compression. When needed, resuscitation was initiated by the treating physician only in three (17%) cases and the rest by delivering midwife or nurse. Convulsions have been observed in 15 patients (83%); 14 of them were treated with anticonvulsant medications.

Table 2 summarizes management at the NICU and the outcomes. Out of the 18 patients admitted to NICU, 15 (83%) patients required respiratory support, and 17 (94%) were sedated by morphine during the cooling procedure. One patient did not receive morphine because of his respiratory depression. Furthermore, 11 (61%) patients received fresh frozen plasma due to coagulation derangement. Seventeen (94%) patients survived to discharge, and all of them were followed in the clinic to the minimum of 1-year of age when this report was written. Due to the unavailability of blood cultures, sepsis was treated empirically. Ten out of the 18 patients received extended-spectrum antibiotics when they had deterioration in clinical and laboratory indicators.

**Table 2:** Nursey management and outcomes

*n*	17
Respiratory support; *n* (%)	15 (83)
• Mechanical ventilation; *n* (%)	10 (56)
• CPAP; *n* (%)	1 (6)
• Nasal cannula; *n* (%)	4 (22)
Duration of mechanical ventilation, days; median (IQR)	4 (3–6)
Sedation/analgesia; *n* (%)	17 (94)
Vasopressors and inotropes; *n* (%)	2 (11)
Extended-spectrum antibiotics; *n* (%)	10 (56)
Duration of antibiotics therapy, days; median (IQR)	10 (7–15)
Fresh frozen plasma; *n* (%)	11 (61)
Age at feeds initiation, days; median (IQR)	5 (4–6)
Age when target feeds reached, days; median (IQR)	7 (6–10)
*Type feeding at discharge:*	
• Breast milk; *n* (%)	13 (71)
• Mixed; *n* (%)	2 (12)
• Formula; *n* (%)	2 (12)
Full oral of feeding at discharge; *n* (%)	17 (100)
Length of hospital stay, days; median (IQR)	12 (9–17)
Survived; *n* (%)	17 (94)
Follow-up of the survived up to 1-year of age; *n* (%)	17 (100)

Abbreviations: CPAP, continuous positive airway pressure; IQR, interquartile range; *n* , number.

Table 3 presents the most important laboratory findings and imaging studies. All survived patients had brain imaging, 4 (22%) had a sonogram, 6 (33%) had a CT scan, and 11 (61%) had an MRI. One patient passed away at the age of 3 days due to pulmonary hemorrhage; therefore, brain CT or MRI was not performed. CT was chosen over MRI in six cases because of financial limitations. All MRI and CT imaging showed evidence of ischemic changes.

**Table 3:** Laboratory and imaging

Highest WBCs, x 10 ^9^ /L; median (IQR)	14.8 (12.8–19.5)
Lowest WBCs, x 10 ^9^ /L; median (IQR)	7.3 (6–9.1)
Lowest platelets, x 10 ^9^ /L; median (IQR)	159 (105–176)
PT, seconds; median (IQR)	25 (20–34)
aPTT, seconds; median (IQR)	36.5 (36–42.)
INR; median (IQR)	2.1 (1.6–2.3)
Admission CRP, mg/L, median (IQR)	2.3 (1.1–10.3)
Highest CRP, mg/L, median (IQR)	21.5 (9.3–42.8)
Admission creatinine, micromole/L, median (IQR)	70.7 (62–80)
Highest creatinine, micromole/L, median (IQR)	80 (62–88.4)
Admission AST, U/L, median (IQR)	80 (47–102)
Highest AST, U/L, median (IQR)	80 (47–102)
Admission ALT, U/L, median (IQR)	40 (21–57)
Highest ALT, U/L, median (IQR)	43 (21–68)
Head sonogram, %	4 (22)
Brain CT, %	6 (33)
Brain MRI, %	11 (61)
Age at brain imaging, days; median (IQR)	9.5 (7–14)

Abbreviations: ALT, alanine aminotransferase; aPTT, activated partial thromboplastin time; AST, aspartate aminotransferase; CRP, C-reactive protein; CT, computed tomography; IQR, interquartile range; INR, international normalized ratio; MRI, magnetic resonance imaging; PT, prothrombin time; WBC, white blood cells.

## Discussion

This report describes as well the difficulties we faced in providing TH in babies with HIE and the solutions we introduced using facilitated cooling techniques with the help of telemedicine. To our knowledge, this is the first report of its kind from an area of conflict. Its implications may promote the use of TH care and improve these infants' outcomes.

The cornerstone of HIE prevention depends heavily on providing early and adequate antenatal care; the incidence rates of HIE in the United Kingdom have decreased over time from 7.6 per 1000 live births in the 1970s to 1.9 in the 1990s. 11 However, in areas where antenatal care is not accessible, these rates are still unduly high; thus, efforts to mitigate HIE sequelae are very much needed. TH, a modality in secondary prevention, is the gold standard of therapy for moderate and severe HIE in high-income countries. 12 This is not exactly the same in LMICs. Controversy about utility and outcomes was strongly challenged by a recent study from four low-income countries. 13

Cooling initiated within the first 6 hours of life appears to be effective in reducing the adverse outcomes. 14 To maintain the core temperature within a target, however, cooling must be well-controlled in a narrow range (33–34°C). 15 To that end, many strategies have been utilized. 5 7 8 9 10 A “facilitated cooling” technique that relies on low-cost devices, such as (Tecotherm-HELIX cooling device), was found effective in a trial from India. 11

Telemedicine has been used globally for many neonatal services, including screening for retinopathy of prematurity, performing tele-echocardiography, newborn resuscitation, and providing family support. 16 Not only has telemedicine been possible, but tele-rounding on NICU patients was found to be a safe and effective approach. 6 The feasibility and efficacy of telemedicine in complicated NICU cases are being explored through an ongoing clinical trial, registered on (ClinicalTrials.gov). 17 In our case, the lack of on-site neonatologists and the logistical difficulties encountered upon transferring critically ill infants to the nearest tertiary center across the borders to Turkey required an innovative approach to these challenges. Specifically, TH requires initiation within 6 hours of birth; therefore, transferring the infant across the border is impractical given time constraints. Thus, initiating TH through telemedicine guidance became the only feasible option to provide appropriate care. This, however, posed new challenges for both the treating team onsite and the neonatology consultants abroad. The providers worked together from admission to discharge. Despite the minimal experience and the paucity of reports to inform the management of such critical cases in the NICU, we were still able to initiate the project and provide appropriate care for our patients.

Unlike in developed countries where high-tech equipment for telemedicine is readily available, we had to settle for a less complicated system and improvise special ways of effective consultation. Treating staff was provided with online classes about HIE treatment protocols and ways to evaluate patients' eligibility for TH. Reviewing the case with the neonatologist and subspecialty consultants through the mobile app (Telegram) and using the HIE calculator to facilitate decisions have smoothened the course of therapy and contributed to a timely adjustment of the plan of care based on the clinical progress and the results of the investigations. Furthermore, applying this therapy through telemedicine reduced the number of cases to be transferred across the border, with the inherent associated risks of this process. More importantly, it enhanced bonding with families and increased parents' satisfaction.

The incidence of HIE in this population is higher than that in developed countries. 1 The lack of effective therapies to mitigate the consequences of HIE provides a great impetus to create and maintain such a project; its implementation, though challenging, was practically within reach. For example, the majority of patients remained within the targeted temperature range (33–34°C) for 84.1% of the cooling duration. This rate is comparable to what is reported by studies conducted in low-income settings. A study from Brazil using low-cost materials for cooling (ice packs) reported staying in the targeted range in 82.3% of the time. 18 Similarly, in a study from India, ice packs cooling maintained temperature within the range in 92.5% of measurements. 19

Implementing TH to reduce mortality and/or morbidity was emphasized in a couple of large clinical trials in high-income countries. 14 20 21 A Cochrane review in 2013 pooled data from 11 pilot studies, mostly from high-income countries, and concluded that findings are in favor of using TH. 22 A meta-analysis published in the same year had pooled data for seven studies from low-income countries 23 and concluded that further studies are required in similar settings. Recently, a large meta-analysis showed overall benefits from TH after pooling 28 studies in different countries. 24 In that study, subgroups analysis revealed possible benefits from TH in LMICs.

Surprisingly, a large clinical trial conducted in India, Sri Lanka, and Bangladesh recommended against TH in LMICs due to increased mortality and morbidity. 13 Of note, almost half of infants in each arm ended up with death or moderate-to-severe neurodevelopmental compromise, and the death rate in the TH group was 42 versus 31% in the control group. Clinical seizures were observed in 74% of patients who received TH and in 73% of those in the control group. Apparently, there is considerable heterogeneity in results from LMICs, specifically in mortality rates. While the TH procedure is well-defined and almost unified among studies in high-income studies, various techniques were used in low- and middle-income ones. However, linking outcomes to the economic status of the origin country may not sound realistic. Furthermore, mortality in patients with HIE can be multifactorial, and care provided is less standardized in those countries. One possible explanation is that HIE may have its start far earlier than the time of birth or the 6-hour window of cooling; since the parturient women in LMIC may attend the medical facilities quite late, rendering TH less effective than it would have been in a healthcare facility in a developed country.

In our study, parameters of telemedicine utilization are promising. On average, the time lapse between seeking consultants' guidance and the initiation of cooling was relatively short, approximately 30 minutes. In most instances, it took the consultant to respond to the help call only 12 minutes, an impressive time frame in such a setting. Decisions of cooling were taken seamlessly and in a timely manner, as the pathway was clear and Calgary tool was available. Clinical findings, despite the facility's limitations, have been promising. We demonstrate that TH usage in managing HIE is feasible through telemedicine. We are unable to produce reliable conclusions in terms of mortality and long-term outcomes beyond the neonatal period, mainly due to a lack of expertise in pediatric development objective assessment, an area where additional efforts are needed. Other limitations of this study include a small sample size and the lack of a control group. Larger studies with robust designs are needed to corroborate our findings and to shed more light on the need of this unprivileged population.

## Conclusions

In limited resources or conflict areas, applying TH to manage HIE is feasible through telemedicine utilizing simple technologies, such as mobile apps. Committed teams of experts along with the treating team in the NICU are a prerequisite for success. This practice seems a promising modality that has the potential to save lives and improve the quality of care in war-ravaged zones.
